# The Book World of Medicine and Science

**Published:** 1900-11-17

**Authors:** 


### THE BOOK WORLD OF MEDICINE AND SCIENCE.

A SHORT PRACTICE OF GYNÆCOLOGY. By HENRY JELLETT, B.A., M.D. Pp. 436. 8vo. Illustrated. (London: J. and A. Churchill. 1900. Price 7s. 6d.)

DR. JELLETT has attempted, not unsuccessfully, in this volume to present a concise account of gynæcological practice which, whilst omitting what is dubious or superfluous, submits most of what is accepted by reliable authorities. Not unnaturally, however, the book is, in fact, an epitome of the practice of Dublin gynæcologists. It is clearly and well written, and illustrated by many woodcuts of a really high order of excellence. The facts are accurately stated, the theory is not unduly expanded, and the suggestions for treatment are eminently judicious. But it cannot be said, nor do we believe Dr. Jellett expects it to be said, that completeness of reference has been obtained. For, in order to keep the book within certain limits of size, anatomical and other prolegomena have not been inserted, historical references are few, and only accepted procedures are described. But, though the tabular mode of fact-presentation adopted with such success in Hart and Barbour's and other textbooks has been utilised, it cannot be said that so much attention has been paid to therapeutical detail. In consequence the book loses much of the value which otherwise, in the eyes of students and general practitioners, would attach to it. Points of interest to English—as opposed to Irish—readers are, that Dr. Jellett advises examination in the recumbent position, and does not mention the Sims speculum. With regard to the ætiology of certain tubal disorders Dr. Jellett does not adopt the extreme views of some as to the frequency of gonococcal infection, and scarcely, we think, gives adequate notice of the notions concerning syphilis which have lately become current in consequence of the work of Shaw-Mackenzie and others. But Dr. Jellett is acutely sensible of the perhaps insufficiently recognised frequency of the occurrence of pelvic tuberculosis. On the whole we must congratulate Dr. Jellett on the manner in which he has performed his task, and we have no doubt of a cordial reception to his work.

FIRST STAGE BOTANY (The Organised Science Series). By A. J. EWART, D.Sc., Ph.D. Pp. 252. (London: W. B. Clive, Strand, W.C. Price 2s.)

THE reviewer's desk nowadays is glutted with a surfeit of little books written, not to teach or tell any new thing; not to place old facts in a new light; not to present even any subject in completeness; but to complete some "series" or other of "little books" designed to fulfil the requirements of some syllabus issued by some "Examining Board." The book at present in our hands professes to teach first-stage botany. But, because the examinees for whom it caters are required to discuss only the flowering plants, this little book is freed entirely from the encumbrance of any reference to thallophyta, bryophyta, or pteridophyta. We are bound to say that Dr. Ewart has done that which he has set himself to do extremely well. His book is well written, the arrangement is admirable, and the many illustrations are excellent. The introductory chapters on the cell, the root, the shoot, and the leaf are as good as anything that has been written in this way; and the author has caught something of the spirit, as well as of the manner, of Huxley. The chapters on classification are no less excellent, and the appendix of technical terms is entirely admirable. But it is no first stage of botany that is taught by this book. One department is taken, and taught briefly, but by no means inadequately. The result is an excellent "cram" book, capable of producing in mind of the tyro no small degree of error in perspective. And we dare wager that no one is more aware of this than Dr. Ewart himself.

TWICE CAPTURED: A RECORD OF ADVENTURE DURING THE BOER WAR. By THE EARL OF ROSSLYN. (Edinburgh and London: William Blackwood and Sons, 1900.)

LORD ROSSLYN went out to South Africa as "roving correspondent" to the "Daily Mail" and the "Sphere" during the Boer war, and he saw a lot. Having put his adventures more or less into shape for the papers, it was not unnatural that he should do the same again in the form of a book, and here it is. It is but an ephemeral work, being too personal, too much tied up with Lord Rosslyn's own adventures, his own impressions, and his own opinions to be of serious importance. But in its own way, as a vivid record of his travels, and as giving series of glimpses into the life of the moment in parts of South Africa which are a good deal off the beaten track, as, for example, in his ride through Basutoland, the book is certainly interesting. There is a good deal in it, however, which had far better not have been said, and at this moment interest is given to this portion of the book by the positive denial given by Lord Roberts to the statements it contains. The book is very well illustrated.

### NOTES ON BOOKS.

JUST as in summer invalids inquire where to bathe, so in autumn they ask where they may best escape the rigours of winter, and to guide them in their choice such books as Dr. Linn's "Health Resorts of Europe" (Henry Kimpton), which is now in its eighth year of publication, is likely to be of much service. It differs somewhat from Bradshaw's "Bathing Places and Climatic Health Resorts" in that while the latter is encyclopædic and touches upon almost every place which in any way poses as a health resort, Dr. Linn's book confines its attention to a smaller number of places. It is nicely written, and gives a good deal of information of many kinds. We have no doubt that its therapeutic index will be conned by many with much anxiety; but we question whether such lists are of much practical service to the uninitiated. To the physician, however, they may sometimes be useful as aids to memory.

THE plan so long adopted by foreign watering places of advertising themselves by the issue of little books descriptive of their charms has of late years been taken up by various health resorts in England, and we can hardly doubt that the Urban District Council of Buxton has done well in distributing among the members of the medical profession an interesting and very nicely illustrated guide book to "Buxton: its History, Waters, Climate, and Scenery" (Henry Blacklock and Co.). Of course everyone knows about the Buxton waters, but in these days when all the world is on the look-out for elevated localities where they can live in pure air above the fogs, many will be glad to be reminded of Buxton, where they can not only enjoy these advantages, but have the comforts of a town. To strong people the country may be all-sufficing, but to the valetudinarian shops and churches, carriages and bath chairs, libraries, and a good postal service, make all the difference between happiness and discontent, and to find all these things a thousand feet or so above sea-level is no small thing.

"THE Humours of a Hydro." By Dagney Major. (Skeffington and Son) describes in a light, but, it must be confessed, not a very interesting manner, some of the oddities and strange characters to be met with at the rather hypothetical establishment which is described in its pages. It does not seem to be a very true picture of anything real, but as a skit it will do for railway reading.

